# An Update of Palmitoylethanolamide and Luteolin Effects in Preclinical and Clinical Studies of Neuroinflammatory Events

**DOI:** 10.3390/antiox9030216

**Published:** 2020-03-05

**Authors:** Marika Cordaro, Salvatore Cuzzocrea, Rosalia Crupi

**Affiliations:** 1 Department of Biomedical and Dental Sciences and Morphofunctional Imaging, University of Messina, Via Consolare Valeria 1, 98100 Messina, Italy; cordarom@unime.it; 2 Department of Chemical, Biological, Pharmaceutical and Environmental Sciences, University of Messina, Via F. Stagno D’Alcontres 31, 98166 Messina, Italy; rcrupi@unime.it; 3 Department of Pharmacology and Physiology, Saint Louis University, St. Louis, MO 63103, USA

**Keywords:** neuroinflammation, clinical, palmitoylethanolamide, luteolin, co-ultramicronization, CNS pathology, adaptive immune response, cell homeostasis

## Abstract

The inflammation process represents of a dynamic series of phenomena that manifest themselves with an intense vascular reaction. Neuroinflammation is a reply from the central nervous system (CNS) and the peripheral nervous system (PNS) to a changed homeostasis. There are two cell systems that mediate this process: the glia of the CNS and the lymphocites, monocytes, and macrophages of the hematopoietic system. In both the peripheral and central nervous systems, neuroinflammation plays an important role in the pathogenesis of neurodegenerative diseases, such as Parkinson’s and Alzheimer’s diseases, and in neuropsychiatric illnesses, such as depression and autism spectrum disorders. The resolution of neuroinflammation is a process that allows for inflamed tissues to return to homeostasis. In this process the important players are represented by lipid mediators. Among the naturally occurring lipid signaling molecules, a prominent role is played by the N-acylethanolamines, namely N-arachidonoylethanolamine and its congener N-palmitoylethanolamine, which is also named palmitoylethanolamide or PEA. PEA possesses a powerful neuroprotective and anti-inflammatory power but has no antioxidant effects per se. For this reason, its co-ultramicronization with the flavonoid luteolin is more efficacious than either molecule alone. Inhibiting or modulating the enzymatic breakdown of PEA represents a complementary therapeutic approach to treating neuroinflammation. The aim of this review is to discuss the role of ultramicronized PEA and co-ultramicronized PEA with luteolin in several neurological diseases using preclinical and clinical approaches.

## 1. Introduction

Pain, redness, increased heat, and swelling are the four cardinal signs of an inflammatory response; loss of function is occasionally added with these four as the fifth mark of an inflammatory response [1]. The “inflammation process” represents the body’s response to different tissue lesions, and as such, involves the recruitment of immune cells and the release of mediators. Consequently, innate and adaptive immune cells, as well as vascular cells and neurons initiate a defense process in order to maintain or restore tissue integrity. Inflammatory events of the central nervous system (CNS) occur at different levels from those of other tissues and involve several types of cells [2,3].

In particular, the first difference relies in the absence of resident dendritic cells in the CNS parenchyma, where perivascular macrophages and vascular pericytes take over the functions of mature dendritic cells in the CNS [4]. As a second feature, the activation of the innate immune cells of the CNS parenchyma, such as astrocytes, microglia, and in some regions, mast cells, may be increase in pathological conditions, such as stroke, trauma, growth of a tumor, or neurodegenerative disease [5,6,7]. In addition, for the body to respond adequately during an inflammatory event, extravasation of the immune cells and inflammatory molecules must take place. These events are indispensable for triggering the immune response and activating the complementary cascade. Nevertheless, in the CNS, the blood–CNS barrier’ reduces the permeability of microvessels, thus making the whole inflammatory reaction more difficult. Only activated T cells may penetrate the barrier, but this does not elicit an efficient reaction to inflammation when compared with that observed in peripheral tissues, where dendritic cells are responsible for the adaptive immune response [8]. Due to these features, it is interesting to point out that the CNS reacts to inflammatory events when these exert a direct effect on the CNS, i.e., in the case of pathogens and tissue damage, and when the inflammatory events are so severe that infiltrating T cells are involved. These observations lead to the introduction of the term “neuroinflammation,” which distinguishes the inflammatory reaction in the CNS from inflammation in different tissues. From this perspective, neuroinflammation is a response of the CNS to a changed homeostasis. There are two cell systems that are able to mediate this response: glia of the CNS, and lymphocytes, monocytes, and macrophages of the hematopoietic system [9]. The neuroinflammation can be triggered by infection, autoimmunity, and toxins, which are defined not just by classical factors, but also by noxious stimuli or psychological stress, such as neurogenic factors. Consequently, the actions promoted by the neuroinflammations are classified as: homeostatic (vasodilation and release of cytokines and neurotrophic factors); maladaptive (release of pro-inflammatory factors); neurotoxic (release of pro-inflammatory factors and breakdown of blood–CNS barrier); and anti-inflammatory (release of pro-inflammatory cytokines, neurotrophic factors, neurotransmitters, and cell adhesion molecules). Neuroinflammation after damage is actively controlled by a complex network of regulatory mechanisms that restrict the potentially harmful effects of persistent inflammation. In particular, chronic, uncontrolled inflammation is characterized by the overexpression of cytokines, such as TNF and IL, reactive oxygen species (ROS), and other inflammatory mediators (such as inducible nitric oxide synthase). All of these signals are identified during injuries to the CNS and are transferred to the injury site by attracting and transporting peripheral macrophages and neutrophils. However, when neuroinflammation is prolonged and macrophage iperactivation is extended, it overwhelms the limits of physiological regulation and causes deleterious effects, including pro-inflammatory signaling pathways, elevated oxidative stress, and death of adjacent neurons that relate to chronic pain pathogenesis, such as neuropathic pain, contributing to neurodegeneration [10,11]. Neuroinflammation is a common mechanism that influences the severity and progression of neurodegenerative disease; for this reason, it is an important target for neuroprotective therapies (Table 1).

## 2. Microglia

Crosstalk between the glia and the neurons, which is necessary for the preservation of homeostasis, is exemplified by the coordination between the immune system and the central nervous system. The glia family is composed of three distinct cell types: microglia, astrocytes, and oligodendrocytes [91].

Both microglia and macrophages, located in the brain and spinal cord parenchyma, represent the key players in the regulation of immunity in the CNS. The production of cytokines and chemokines, as well as the production of free radicals, such as reactive oxygen species (ROS) and nitric oxide (NO) known to contribute to neuronal and tissue damage, are implicated in the regulation of neuroinflammation.

Furthermore, microglia activation, which is important in the development of neurogenesis, plays a crucial role in both synaptic pruning and damage restoration by removing apoptotic cells and secreting growth factors [92]. It is then clear that glia cells are not just cells that fill “the space not occupied by neurons,” as Virchow suggested in the late nineteenth century, but dynamically play a role in neuronal support and dysfunction [93]. Microglia comprise only 10% of the total cell population of the brain, but represent the major resident immune cells that survey the microenvironment looking for cellular debris and pathogens, and produce factors, such as cytokines, that influence the surrounding astrocytes and neurons. Meanwhile, microglia cells are classified as good players because remove cellular debris via phagocytosis, release neurotrophic factors and anti-inflammatory cytokines, prevent neuronal injury, restore tissue integrity in the injured brain, maintain the plasticity in the neuronal circuits, and encourage the protection and remodeling of synapses [94,95]. Structurally, microglia show a dynamic and active phenotype with ongoing retraction and extension of processes in the brain’s structure [96]. Following insults that are not due to pathogenic agents, tissue damage, or exposure to neurotoxic substances, microglia are activated by stimulating the inflammatory response, which further involves the immune system. 

Microglia can act via specialized pro-resolving lipid mediators (SPMs) receptors, cannabinoid receptors 2 (CB2), and aryl hydrocarbon receptors (AHR) in response to their ligands, such as SPMs; cannabinoids; and gut-derived metabolites, such as tryptophan (TRP). Activation of these microglial receptors enhances the phagocytic activity, reduces the expression of pro-inflammatory mediators, and increases the production of anti-inflammatory mediators and SPMs, thus promoting the resolution of neuroinflammation and pathological pain [11]. 

Following this activation, pro- and anti-inflammatory cytokines are released in the cerebral parenchyma. There are two phenotypes that describe this dichotomy at the level of the microglial phenotype: M1 and M2. The microglial phenotype changes in relation to the insult, the extent of the damage, and the post-injury recovery [97,98].

For example, lipopolysaccharide (LPS) and the pro-inflammatory cytokine interferon γ (IFNγ) promote a “classically activated” M1 phenotype, which produces high levels of pro-inflammatory cytokines and oxidative metabolites that are essential for host defense and phagocytic activity, but that also cause damage to healthy cells and tissue. Conversely, activating microglia in the presence of cytokines, such as IL-4 or IL-13, promote an “alternatively activated” M2 phenotype [99,100]. Although there is limited data on resident M2 microglia in the brain, it is thought that much like the M2 macrophages, these cells can promote angiogenesis, wound healing and tissue repair, extracellular matrix remodeling, and suppress destructive immune responses [101]. M2 microglia express specific antigens, such as arginase 1 (Arg1); mannose receptor (MRC), found in the inflammatory zone 1 (FIZZ1); and chitinase 3-like 3 (Ym1), among others. Furthermore, cultured microglia exposed to IL-4 or IL-13 develop the M2 phenotype, which can result in extensive neurite elongation and outgrowth across inhibitory surfaces in in vitro co-culture systems [99,102,103,104]. 

In the human brain, microglial activation and neuroinflammation have been associated with viral or bacterial infection, autoimmune diseases, head trauma, vascular system damage, neuropsychiatric disorders, and neurodegenerative illness.

## 3. Astroglia

Astrocytes are classified as immunocompetent cells that are good regulators of brain inflammation and can be divided into two major groups: protoplasmic astrocytes (gray matter, type-1) and fibrous astrocytes (white matter, type-2). Protoplasmic astrocytes, which are widely distributed in gray matter, have a larger size (≈50 μm) and more organelles than fibrous astrocytes, and at least one process contacts blood vessels through perivascular endfeet, as well as forming multiple contacts with neurons. These astrocytes regulate local blood flow and neuronal homeostasis [105,106]. Fibrous astrocytes originate from radial glial cells that are capable of differentiating neurons, astrocytes, and oligodendrocytes during brain development, and these astrocytes highly express glial fibrillary acidic protein (GFAP), nestin, and vimentin [107,108]. Although the specific function remains to be characterized, fibrous astrocytes also contact vessel capillaries like the protoplasmic astrocytes [109]. Generally, elevated GFAP is a common feature of the activation state of astrocytes.

Like microglia, astrocytes can become activated through a process known as astrogliosis. This process is characterized by altered gene expression, hypertrophy, and proliferation [110]. Because of their structure, astrocytes are defined as “territorial cells” with an essential role in the encouraging of cells to provide neurons for homeostasis [111]. Astrocytes strictly cooperate with the surrounding structures in the nervous system and provide the regulation of their functions. One of the most vital roles of astrocytes at the synapse is the clearance of neurotransmitters. For example, the astrocytic processes associated with excitatory synapses are enclosed with glutamate transporters, which maintain a low ambient glutamate level in the CNS and allow for glutamate receptor activation at synapses [112]. Nonetheless, astrocytes form the glia limitans around blood vessels, preventing the entry of immune cells via the blood–brain barrier (BBB) into the CNS parenchyma. They are characterized by the augmented expression of glial fibrillary acidic protein (GFAP) and signs of functional deficiency [113]. Astrocytes are able to release interleukins, NO, cytokines, and other cytotoxic molecules that are able to exert either neuroprotective or neurotoxic effects [114]. In particular, recent studies have shown two different reactive astroglia populations in the adult CNS: A1 and A2. A1 astrocytes can aggravate disease pathogenesis and destroy both neurons and oligodendrocytes. In fact, in several neurodegenerative diseases, A1 astroglia are indeed present in mouse and human, particularly around areas of disease pathology. Vice versa, A2 astrocytes seem to upregulate neurotrophic genes that encourage neuronal survival. Additionally, activation of both A1 and A2 astroglia is a result of crosstalk between activated microglia and astroglia in diseases [115,116,117]. It is very likely that the astrocyte immune activation is the result of a very complex interaction between the pro- and anti-inflammatory process, and the neurotoxic and neurotrophic processes. The two main characteristics of astrocytes are their elaborated intracellular calcium signaling, also termed calcium waves, and their high degree of intercellular communication, which is mediated by gap junctional channels (GJCs) [118,119]. Connexins (Cx) are the molecular constituent of GJC, where Cx43 is the major astrocytic connexin identified. However, this does not exclude the possibility that other astrocytic connexins could be expressed, either in specific brain regions or in pathological situations [118,119]. 

## 4. Oligodendroglia

In the central nervous system, myelin is made up of cells called oligodendrocytes. Such cells are important in propagating action potentials along axons; an important duck function they perform is the support they give to neurons by producing neurotrophic factors. However, these cells are very vulnerable to both oxidative stress and the toxicity caused by an excess of glutamate. The oligodendroglia cells are characterized by the presence of a large pool of iron, but despite this, they have a low capacity for anti-oxidative mechanisms, which render the cells especially sensitive to oxidative stress [120]. The crosstalk between microglia and oligodendrocytes regulates cerebral homeostasis. After being activated at the microglia, it produces the pro-inflammatory mediators [121]; they are essential for killing pathogens, but on the other hand, these mediators can damage both the glia and adjacent neurons. Under these conditions, oligodendrocytes are particularly susceptible to microglial factors due to their high metabolic activity and their energy needs.

Its response is represented by the production of poor-quality myelin, which may stimulate the pathology detected in many neurological diseases. Oligodendrocytes, in particular oligodendrocyte progenitor cells (OPCs), express receptors for fibroblast growth factor (FGF), epidermal growth factor (EGF), platelet-derived growth factor (PDGF), insulin-like growth factor 1 (IGF-1), and vascular endothelial growth factor (VEGF). Recent studies demonstrate that VEGF plays a key role in the remyelination process by promoting the migration of OPCs, despite being considered one of the most important factors in the regulation of angiogenesis [122]. Moreover, oligodendrocytes cell surfaces have receptors for a wide-ranging of mediators, such as IL-4, IL-6, IL-7, IL-10, IL-11, IL-12, and IL-18 [123]. Recent evidence also demonstrates that oligodendrocyte also express antigen-presenting molecules and co-stimulatory molecules, complement and complement receptor molecules, complement regulatory molecules, tetraspanins, neuroimmune regulatory proteins, extracellular matrix proteins, and many others [124]. Necrosis and apoptosis are events that can activate oligodendrocytes in various ways in the central nervous system. In relation to the activated path, oligodendrocytes can also release factors that stimulate microglia; it is still possible that stressed cells can trigger the protective pro-repair responses of microglia.

## 5. Mast Cells

Mast cells, also called effector cells, are first responders that are able to intervene as catalysts and recruiters in initiating, amplifying, and prolonging all the immune and nervous responses that arise from their activation. They originate from a hematopoietic lineage and are involved in a number of normal physiologic functions, such as immunity [125], angiogenesis, and tissue remodeling, as well as being implicated in multiple pathologic conditions [126]. Their purpose is to produce various mediators, such as cytokines, lipid metabolites, enzymes, biogenic amines, growth factors, NO, ATP, heparin, and neuropeptides [127]. It is important to consider that the release of the mediator through the degranulation process is very rapid, whereas the activation is more long lasting, allowing the release of newly formed mediators [128]. Under basal conditions, without disease, trauma, or stressful events, the number of mast cells is lower than that of neurons, microglia, and other cells residing in the brain. Even if in a limited number of the activated mast cells are able to influence the BBB, neurons, astrocytes, and microglia, there is a considerable increase in mast cell degranulation after stroke in the immature brain, as well as after a transient global ischemia in adult rats, or even after the deprivation of in vitro oxygen glucose; all these events imply that mast cells really play a role in determining neuronal damage [129,130,131]. There are numerous molecular mechanisms that have been identified as determining the potential interactions between mast cells and microglia [5]. It has been shown that the activation of P2 receptors on microglia by ATP stimulates the release of IL-33, which after binding to mast cell receptors, triggers the release of IL-6, IL-13, and chemo-attractive monocytes 1; in turn, these molecules are able to regulate the activity of the microglia. Likewise, mast cell tryptide activates microglia-activated receptor 2 (PAR2) receptors, and promotes the release of pro-inflammatory mediators, such as TNF-α, IL-6, and ROS, which in turn upregulate the expression of PAR2 receptors on mast cells [132]. Activated mast cells upregulate P2X purinergic receptors, ligand-ions, PR2X4 microglia receptors, promoting brain-derived neurotrophic factor (BDNF) [133]. Mast cells react dynamically with astrocytes like microglia. Both mast cells and astrocytes are co-located in both perivascularization and in the thalamus [134]. In vitro studies have shown that mast cells activate astrocytes through activation of CD40–CD40 ligand interactions, and are stimulated to release cytokines, leukotrienes, and histamine [134]. Interestingly, astrocytes also have histamine receptors (H1R and H2R) [135], and cytokines released from astrocytes can induce mast cell degranulation [136].

## 6. Mediators during Neuroinflammation: Inflammasome, Cytokines, and Chemokines

Inflammasome, cytokines, and chemokines play an important role in mediating neuroinflammation; for this reason, they have received a considerable amount of attention as possible therapeutic targets [137,138]. Inflammasomes are cytosolic multiprotein complexes that upon assembly, activate the pro-inflammatory caspase-1, which is responsible for the maturation and secretion of the inflammatory cytokines IL-1β, IL-18, and IL-33, as well as the induction of the pyroptosis process [138,139]. These pro-inflammatory cytokines have been shown to promote a variety of innate immune processes linked to infection, inflammation, and autoimmunity, and thus play an instrumental role in instigating neuroinflammation during old age and the eventual occurrence of cognitive impairment, neurodegenerative diseases, and dementia [139]. Everything we know about the inflammasome activation and its role in CNS inflammation is still incomplete and is principally based on in vitro studies with primary microglia and microglial cell lines, and in vivo studies with transgenic KO mice that lack the expression of specific inflammasome components throughout the body [138].

In addition to microglia and astrocytes, cytokines are large proteins (15–25 kDa) that are mainly released from immune cells, such as monocytes, macrophages, and lymphocytes. Cytokines are stimulated in circumstances where inflammation, infection, and/or immunological changes occur, and are specifically involved in tissue repair and homeostasis restoration [140,141]. Cytokines are an exceptionally large and diverse group of pro- or anti-inflammatory factors that are grouped into families based upon their structural homology or that of their receptors [137]. Interleukin-1β (IL-1β), interleukin-6 (IL-6), and tumor necrosis factor-α (TNF-α) are among the most widely investigated pro-inflammatory cytokines, whereas interleukin-4 (IL-4) and interleukin-10 (IL-10) are well-known anti-inflammatory cytokines. 

Chemokines are a category of secreted proteins in the family of cytokines whose basic role is to cause cell migration [142,143]. These “chemotactic cytokines” include the chemoattraction of leukocytes and the flow of immune cells to locations throughout the body [137]. The two principal chemokine ligand superfamiles are named according to the arrangement of the (typically four) cytokines within them: in the CC family, the first two cysteines near the amino terminus are adjacent, whereas in the CXC family, there is one amino acid between them [144].

In addition to being essential for the synchronization of immune responses throughout the body, cytokines and chemokines are implicated in the control of CNS–immune system interactions. They are produced primarily not only by white blood cells or leukocytes, but also by a variety of other cells as a response to various stimuli under both pathological and physiological conditions. In the nervous system, cytokines and chemokines function as neuromodulators and regulate neurodevelopment, neuroinflammation, and synaptic transmission [137]. Cytokines and chemokines are essential to the immune function of the brain, helping to maintain immune surveillance, promote leukocyte traffic, and employ other inflammatory factors [145]. During neuroinflammation, immune cells and cells of the nervous system can release cytokines and chemokines, as well as respond to them by way of cytokine and chemokine receptors [146,147,148]. 

## 7. Resolution of Neuroinflammation

Inflammatory states may activate a program of resolution that involves the production of lipid mediators with the capacity to switch off inflammatory processes [149]. Several studies have shown that neuroinflammatory processes are noticeably dangerous for the body; as such, numerous studies have been performed, and others are ongoing, to develop new therapeutic strategies. However, to do this, it is necessary to know not only about the neuronal and non-neuronal cells, but also their mechanism of action. The resolution of neuroinflammation is a process that allows for inflamed tissues to return to homeostasis. In this process the important players are the lipid mediators (LM).

Lipid mediators are widely appreciated for their important roles in initiating the leukocyte traffic required in the host defense [150]. These include the classic eicosanoids, prostaglandins (PGs), and leukotrienes (LTs) [151,152] that stimulate blood flow changes, edema, and neutrophil influx to tissues [153]. Novel resolution-phase mediators that possess potent proresolving actions were identified and named resolvins, protectins, and maresins.

Biochemically, lipid mediators represent a diverse family of endogenous bioactive molecules that are enzymatically derived from fatty acid substrates. Prostaglandins, a family of extensively studied lipid mediators, are synthesized from arachidonic acid and are elevated after an acquired neurological injury, such as a traumatic brain injury (TBI). The first LMs synthesized are prostaglandins and leukotrienes, which are generated to activate and amplify the cardinal signs of inflammation. In particular, prostaglandins E2 and D2 induce production of mediators (lipoxins [154], resolvins, and protectins [155]) with both anti-inflammatory and pro-resolution activities. These mediators are classified as endogenous pro-resolution molecules that are not immunosuppressive. SPMs are able to stimulate and accelerate resolution via mechanisms that are multi-factorial. On this basis, resolution is not a passive process, but conversely is an active and dynamic process that is orchestrated by distinct cellular events and endogenous chemical mediators. In this context, prostaglandins are considered LMs that can promote the neuroinflammation process. In an acute inflammatory response, prostaglandins regulate local changes in blood flow and pain sensitization. 

**Figure 1:**
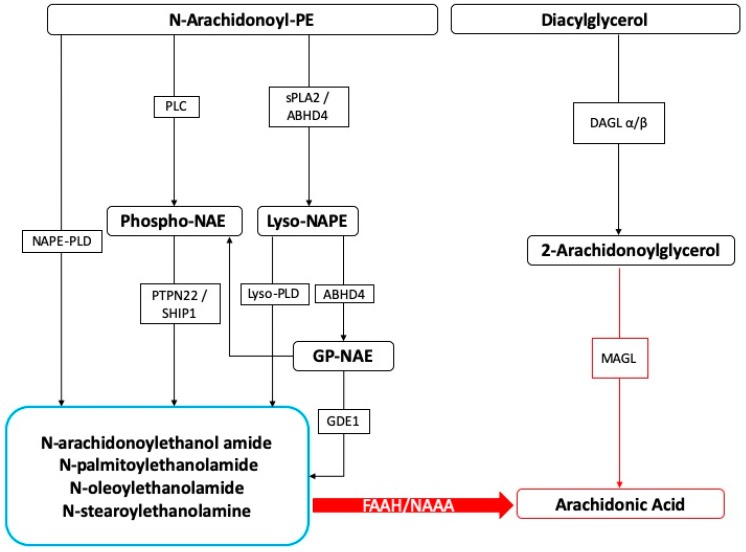
Fatty acid ethanolamines’ metabolism and catabolism. Abbreviations: ABHD: α/β-Hydrolase domain containing, DAGL: diacylglycerol lipase, FAAH: fatty acid amide hydrolase, GDE: glycerophosphodiesterase, MAGL: monoacylglycerol lipase, NAAH: N-Acyl-ethanolamine-hydrolyzing acid amidase, NAPE: N-acyl-phosphatidylethanolamine, PLC: phospholipase C, PLD: phospholipase D, PTPN22: tyrosine phosphatase, SHIP1: inositol 5′-phosphatase, sPLA2: secretory phospholipase A2.

On the other hand, SPMs also show beneficial effects in different neuroinflammation models, including stroke, Alzheimer’s disease (AD), spinal cord injury, and neuropathic pain [156,157,158,159,160]. In order to neutralize the chronic inflammatory processes, a resolution program based on the production of lipid mediators is necessary and therefore able to block inflammation. In the presence of chronic inflammatory conditions, several mechanisms of resolution are triggered, and among these, the production of lipid mediators is known to extinguish inflammation [161]. The existence of molecules involved in endogenous protective mechanisms that are activated in the body due to severe tissue damage or the stimulation of inflammatory responses and nociceptive fibers is well recognized. In this context, we believe the N-acylethanolamines, a class of naturally occurring lipidic mediators, are constituted of a fatty acid and ethanolamine, namely the fatty acid ethanolamines (FAEs). The main FAE family members include the endocannabinoid N-arachidonoylethanolamine (anandamide or 5Z,8Z,11Z,14Z)-N-(2-hydroxyethyl)icosa-5,8,11,14-tetraenamide), and its congeners N-stearoylethanolamine (N-(2-hydroxyethyl)stearamide), N-oleoylethanolamine (N-2-hydroxyethyl-9(Z)-octadecenamide), and palmitoylethanolamide (PEA) (Figure 1).

## 8. PEA: Mechanism of Action

N-acylethanolamines are classified as naturally occurring lipid mediator molecules composed of a fatty acid and ethanolamine, and are collectively identified as “fatty acid ethanolamines” (FAEs). These are endogenous molecules that have the characteristic of being involved in various mechanisms of endogenous protection, which are activated in the body by different types of tissue damage or stimulation of inflammatory responses and nociceptive fibers. The members of the FAE family are the endocannabinoid N-arachidonoylethanolamine (anandamide, or 5Z,8Z,11Z,14Z)-N- (2-hydroxyethyl)icosa-5,8,11,14-tetraenamide) and its congeners N-stearoylethanolamine (N-(2-hydroxyethyl)-stearamide), N-oleoylethanolamine (N-2-hydroxyethyl-9(Z)-octadecenamide), and PEA. 

PEA and its congeners are constituted from N-acylated phosphatidylethanolamine (NAPE) via different enzymatic pathways [162], the principal one producing a membrane-associated NAPE-phospholipase D, which further produces the respective NAE and phosphatidic acid. This enzyme is able to convert N-palmitoyl-phosphatidyl-ethanolamine into PEA. In the mammalian brain, NAEs are hydrolyzed by (1) fatty acid amide hydrolase in the endoplasmic reticulum, which breaks down NAEs into the corresponding fatty acid and ethanolamine, and (2) lysosomal NAE-hydrolyzing acid amidase (NAAA) [163]. NAAA is found mainly in macrophages, as well as in the brain, where it hydrolyses NAEs with less than 18 carbon atoms, i.e., PEA, but not N-oleoylethanolamine and N-stearoylethanolamine [16,164]. 

In contrast, fatty acid amide hydrolase hydrolyses all three NAEs. PEA is abundant in mammals; there is evidence for the presence of PEA, as well as other FAEs, in marine species and sea urchin ovaries [165]. The regional distribution in the rat brain of orally administered PEA (≈100 mg·kg^−1^) has been investigated through the use of N-[9-3H]-PEA by Artamonov and Gabrielsson [166,167]. The authors found that N-[9,10-3H]-PEA mainly accumulated in the hypothalamus, pituitary, and adrenal glands 20 min after oral administration [166]. The presence of the labelled PEA in the brain (≈98 ng·mg^−1^ of brain tissue) demonstrated the ability of the compound to penetrate, although in small amounts, through the blood–brain barrier [166].

Mechanistically, PEA may be a ligand for peroxisome proliferator activated receptor α (PPARα), one of a group of nuclear receptor proteins that function as transcription factors regulating the expression of genes. In particular, the α- and γ-isoforms of PPAR are associated with pro-inflammatory effects. Moreover, in PPARα null mice or mice with blocking PPARα antagonists, the anti-inflammatory, anti-nociceptive/anti-neuropathic, and neuroprotective effect of PEA was not detected [168]. PEA is produced through an “on-demand” synthesis within the lipid bilayer, where N-phosphatidylethanolamine-specific phospholipase D (NAPE-PLD) releases it from its membrane precursor, N-palmitoylphosphatidylethanolamine, but it is also present in “homeostatic” conditions [169].

An “entourage effect” was also proposed to explain PEA’s pharmacological activities regarding improving the anti-inflammatory and anti-nociceptive function of other endogenous substances through potentiating their receptor binding or inhibiting metabolic degradation. Both anandamide and its congeners, such as PEA, have the type 1 transient vanilloid receptor (TRPV1) in common, whose function can be stimulated by harmful heat, low pH, and capsaicin. Moreover, anandamide itself is a TRPV1 receptor agonist, and PEA can enhance anandamide stimulation of the human TRPV1 receptor in a cannabinoid CB2 receptor antagonist-sensitive fashion, which could be considered as PEA acting indirectly by potentiating the actions of anandamide. Mast cells and microglia reportedly express TRPV1 receptors [170].

PEA also has an affinity to cannabinoid-like G-coupled receptors GPR55 and GPR119 [171]. The GPR55 receptor is widely expressed, and therefore its activity is correlated with multiple physiological processes. In particular, GPR55 is expressed in the frontal cortex, striatum, hippocampus, hypothalamus, cerebellum, brainstem, and microglia [172,173,174]. Balenga and colleagues showed that GPR55 activity modulates RhoA-dependent neutrophil migration, and it may prevent oxidative damage.

GPRl19 is expressed mainly in the pancreas and gut, in particular in β cells, where its activity modulates glucose-dependent insulin secretion, as well as in enteroendocrine L-cells, where it regulates the secretion of glucagon-like peptide 1 [175,176]. In normal-weight and healthy patients, it was observed that gut GPR119 expression rapidly increased following acute fat exposure, thus suggesting a potential involvement of GPR119 in type 2 diabetes, metabolic disorders, and obesity. 

The main endogenous ligands of GPR119 are OEA, PEA, and AEA [177,178,179]. 

## 9. PEA: Role in Pathological and Physiological Conditions

**Figure 2:**
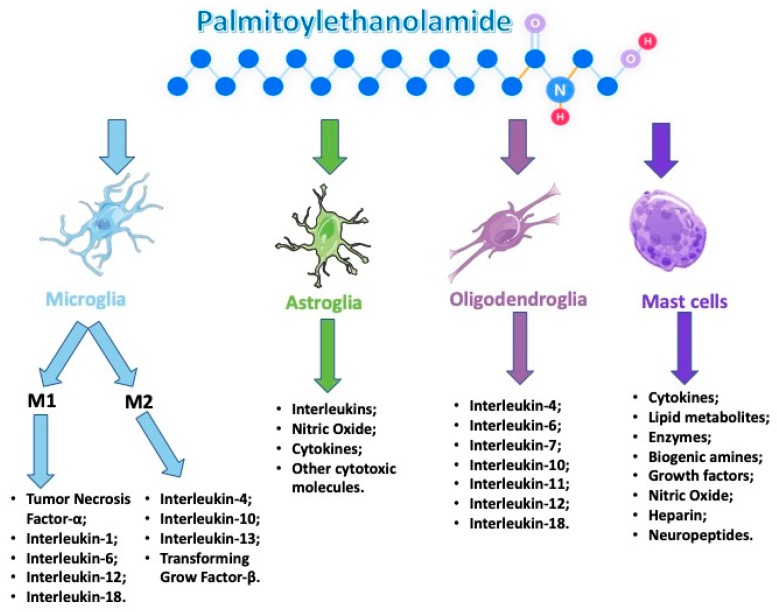
Palmitoylethanolamide (PEA) acts on several types of cells that are involved during an neuroinflammation event.

Several reports showed a role of PEA in maintaining cellular homeostasis by acting as a mediator of the resolution of inflammatory processes. PEA is synthesized/metabolized by both microglia and mast cells. Moreover, it down-modulates mast cell and microglia activation, and very interesting tissue levels of PEA are augmented in brain regions that are implicated in nociception and in the spinal cord following the induction of neuropathic pain and other conditions related to strokes (Figure 2). 

In particular, endogenous levels of PEA are altered in pathological conditions accompanied by cell damage and inflammatory processes. In inflamed tissues, for example following tissue ischemia, the levels of PEA increase significantly [180,181]. Increased PEA levels have also been observed in the brain tissues of animals in a state similar to multiple sclerosis and following a spinal cord injury [182,183]. At the cutaneous level, increases in PEA levels have been observed in animals affected by diabetic neuropathy, after exposure to irritants, and in the case of atopic dermatitis [184,185,186]. In a pain model, PEA inhibits peripheral inflammation and mast cell degranulation, as well as exerts neuroprotective and antinociceptive effects; these actions are accompanied by a decrease in NO production, neutrophil influx, and the expression of proinflammatory proteins, such as iNOS [187]. Moreover, PEA protects endothelial function from oxidative and inflammatory injuries. PEA administration improves neurological, emotional, and biochemical outcomes following TBI by ameliorating the secondary injury components of TBI, reducing both the infiltration and activation of mast cells [188]. It was demonstrated that in mice subjected to a compression model of spinal cord trauma, PEA administered systemically at 6- and 12-h post-injury considerably diminished the severity of spinal cord trauma via the reduction both mast cell activation and infiltration [189]. Furthermore, another study reported that intraperitoneal administration of PEA is able to reduce tissue injury and spinal cord inflammation, nitrotyrosine formation, neutrophil infiltration, pro-inflammatory cytokine expression, nuclear transcription factor kB activation, and inducible nitric oxide synthase expression and apoptosis, as well as ameliorate the recovery of motor limb function [190]. In several preclinical studies on intestinal inflammation, it was reported that PEA improves the symptoms of colitis in mice [191,192]

All these data indicate that the alterations of the levels of PEA in the conditions accompanied by inflammatory processes suggest that the exogenous contribution of the molecule may favor or accelerate the process of resolution of inflammation and the restoration of tissue homeostasis (Table 2).

In neurological diseases, increases in PEA levels were observed in the patients liquor with schizophrenia, chronic migraine, and multiple sclerosis [200,201,202]. In multiple sclerosis, the levels of PEA also increased in plasma [203]. Orefice et al. reported a significant increase of PEA, anandamide, and oleoylethanolamide plasma levels in patients [204].

At the abdomino-pelvic level, PEA levels increase in the duodenum of celiac patients and in the colon of patients with colitis ulcerative [184,205].

On the other hand, there exist some pathological situations in which the levels of PEA are dramatically decreased. This happens, for example, in the synovial fluid of patients with arthritis or rheumatoid arthritis, in which the levels of PEA compared to subjects without joint pathologies are about a thousand times lower [206]. 

Also some clinical studies demonstrated the effect of PEA on the pain condition; in particular, Andresen et al. reported on a ultramicronized PEA that has good safety and tolerability in the treatment of patients with spinal cord injury [207]. In these studies, PEA represents a promising supplement to therapeutic management for neuropathic pain, with the potential for good tolerability and a low susceptibility for side effects [208,209,210,211,212,213,214].

## 10. PEA: Ultramicronization Process

The therapeutic use of PEA is strictly dependent on its lipophilic nature; on this basis, PEA is insoluble in water and poorly soluble in most other aqueous solvents, with the logarithm of its partition coefficient (log P) being greater than 5 [215]. 

PEA is usually administered via a parenteral route, but in particular in humans, oral treatment represents the goal standard due to patient compliance, versatility, and ease. Structurally, PEA is formed using a heterogeneous particle size, and for these reasons, presents some limitations in bioavailability and solubility. Due to these considerations, the oral absorption of PEA is strongly limited by the dissolution rate, with the amount absorbed conceivably showing an inverse relation to particle size [216]. A possible approach to bypass this problem is represented using micronization. The micronization process is applied both to reduce the particle size and to improve the bioavailability and effectiveness of particularly low-water-soluble molecules, thereby increasing the dissolution rate [217,218,219]. 

Both micronized and ultramicronized PEA oral administration showed superior pharmacological action in inflammatory pain induced by carrageenan, in contrast to a non-micronized PEA. Based on this, several studies were performed with these formulations; in fact, micronized pharmaceutical-grade formulations of PEA acquired via a jet milling process are now employed in both human and veterinary medicine to study inflammatory, hyperalgesic, and allergic disorders [220,221,222].

Similar to naïve PEA (particle size profile ranging between 100 and 700 µm), micronized and ultramicronized PEA showed a different particle size profile (2–10 µm and 0.8–6 µm at most, respectively). Micronization and ultramicronization processes yield a different crystalline structure with a higher energy content and smaller particle size, which result in better diffusion and distribution of these molecules compared to the naïve form, and thus superior biological efficacy [220,222]. The pharmacological profile of PEA and the pre-clinical results have inspired a series of clinical studies that have used PEA in the micronized and ultramicronized forms, where they predominantly focused on the pathological conditions associated with neuroinflammation and chronic and/or neuropathic pain [220,223]. In particular, a multicenter, double-blind, randomized study controlled with two doses of PEA versus placebo showed the analgesic dose-dependent effect of micronized PEA (PEA-m) in patients affected by lombosciatalgia with a compressive origin [211]. In the same conditions, therapy with PEA-m allows for a considerable reduction of the use of nonsteroidal anti-inflammatory drugs NSAIDs, suggesting the possibility of simultaneous use in order to reduce the use of NSAIDs, which if taken for long periods, can induce significant side effects [210]. The results obtained in patients with low back pain have also been confirmed on a large number of patients with different chronic pain pathological conditions, such as radiculopathy, osteoarthritis, joint pain, tibia fracture, spinal surgical failure, post herpetic neuropathy, diabetic neuropathy, and oncological pain [197,199,209,212,213] (Table 3). 

Moreover, Evangelista et al. reported that in patients affected by carpal tunnel syndrome, the oral administration of ultramicronized PEA improved sleep quality, verifying a correlation between sleep disorders and pain intensity [208]. PEA-m, in a preclinical study conducted by Crupi et al., used for the first time as a pre-treatment, showed a significantly neuroprotection after 1-methyl-4-phenyl-1,2,3,6-tetrahydropyridine (MPTP) intoxication in mice by protecting not only against loss of TH+ neurons, but also the alterations of the dopamine active transporter (DAT), α-synuclein, and β3-tubulin in the substantia nigra. Moreover, pre-treatment with PEA-m showed an important reduction in the MPTP-induced proinflammatory cytokine expression and showed a pro-neurogenic effect in the hippocampus. These data support the idea of PEA-m as a potential therapeutic candidate to prevent dopamine neuronal cell degeneration in the pathogenesis of Parkinson’s disease [193,224].

The data, obtained with ultramicronized PEA, showed that it was also able to decrease all the inflammatory pathways that were activated during pulmonary fibrosis, as well as dermatitis, myocardial ischemia, or contrast-agent-induced nephropathy [194,195,196,197]. Furthermore, Scuderi et al. showed that PEA-um was a novel and effective promising treatment for AD with the potential to be integrated into a multitargeted treatment strategy in combination with other drugs [198]. Finally, the association of PEA-um with standard neuropathic pain medications, such as pregabalin and oxycodone, allowed for highlighting analgesic pharmacological effects with very low doses of these drugs, suggesting an additive or synergistic effect among neuropathic pain medications acting on neuronal cells with PEA, which acts predominantly on non-neuronal cells [225,226] (Table 3).

## 11. Luteolin

Several studies support the association between inflammation; neurodegenerative diseases; oxidative stress; neuropsychological disorders, such as anxiety and depression; and mild cognitive disorder [227,228,229,230]. Flavonoids have been shown to display many neuroprotective and inflammatory actions [208,231]. Among the large family of flavonoids, luteolin has a good spectrum of action. Luteolin (3′,4′,5,7-tetrahydroxyflavone) is a common flavonoid in many fruits, vegetables, and medicinal herbs [232]. Chemical data show that luteolin has a C6-C3-C6 structure with two benzene rings and one oxygen-containing ring with a C2-C3 carbon double bond [233,234]. Structure–activity studies have revealed that the presence of hydroxyl moieties at carbons 5, 7, 3′, and 4′ positions of the luteolin structure, and the presence of the 2–3 double bond, are responsible for its multiple pharmacological effects [233]. Luteolin represents one of the most powerful and effective polyphenols, which has displayed numerous biological properties, such as antioxidant, anticancer, anti-inflammatory, and neuroprotective properties in in vitro and in vivo models [233,235,236,237,238,239,240,241]. It was formerly supposed that the oral bioavailability of flavonoids was very low. Nevertheless, Zhou et al. studied the bioavailability of luteolin in peanut hull extracts (PHEs) [242]. In particular, the research reported that the efficient permeability (P_eff_) and absorption rate constant (k_a_) of pure luteolin (5.0 μg/mL) were not significantly dissimilar in both the duodenum and jejunum, but notably greater in the colon and ileum. Pharmacokinetics analysis after subsequent oral administration of a single dose of pure luteolin (14.3 mg/kg) or PHE (containing 14.3 mg/kg of luteolin) in rats displayed that the peak concentration of luteolin in plasma was 1.97 ± 0.15 μg/mL for luteolin and 8.34 ± 0.98 μg/mL for PHE. Luteolin seems to be inertly absorbed in the rat intestine [242]. 

Moreover, luteolin has been revealed to inhibit cytokine expression, nuclear factor kappa B (NF-kB) signaling, and TLR4 signaling at micromolar concentrations in immune cells, such as mast cells [243,244,245]. Additionally, the ability of luteolin to inhibit the Keap1-Nrf2-ARE pathway in PC12 cells was demonstrated [246]. Numerous studies have shown that luteolin can improve the neurotoxic effect of Aβ fragment 25–35 (Aβ_25–35_) in murine cortical neurons [247], as well as increase locomotor and muscular alteration in mice exposed to MPTP [248]. Moreover, luteolin has been used for the treatment of multiple sclerosis (MS) due to its inhibition of activated peripheral blood leukocytes that have been isolated from MS patients [249,250]. 

Luteolin can reduce experimental allergic encephalomyelitis (EAE), which is now employed as a murine model to analyze MS [251]. Luteolin treatment has also displayed additive effects in regulating cell proliferation and the production of pro-inflammatory cytokines, as well as the ratio of the cell migration mediator matrix metallopeptidase-9 (MMP-9) and its inhibitor called tissue inhibitor of metalloproteinase (TIMP-1) [249]. In this context, this suggests that it is reasonable to consider luteolin as a valuable adjuvant to IFN-β for the management of MS.

Another work by Xu et al. showed that treatment with luteolin can decrease secondary brain injury provoked by traumatic brain injury (TBI), involving brain water content, neuronal apoptosis, and neurological deficits [252]. 

A clinical study containing fifty children (4–10 years old; 42 boys and 8 girls) with autism spectrum disorder (ASD) described that there was an important improvement in adaptive functioning (as measured using the Vineland Adaptive Behavioral Scales domains) following a combination of luteolin (100 mg/capsule) and quercetin (70 mg/capsule) for 26 weeks in dietary supplementation [253]. Likewise, a significant reduction was also reported after stratification for age, sex, and history of allergies in the Aberrant Behaviour Checklist subscale scores.

## 12. Co-Ultra PEA + Luteolin

The pharmacodynamic properties of PEA and those of luteolin appear complementary, suggesting that if administered in combination, they can fight the two main conspirators of chronic diseases: low-grade inflammation and oxidative stress. Confirming this hypothesis, many studies have shown that joint treatment using PEA plus luteolin has a superior effect compared to the molecules used alone by stimulating both hippocampal neurogenesis and dendritic spine maturation [254,255]. These studies have also shown that the pharmacological properties of PEA + luteolin (PEALut) become synergistic when PEA and luteolin were simultaneously submitted to the micronization process with a jet mill technique in a mass ratio of 10:1. This is probably due to the fact that PEA is poorly soluble in aqueous media and is difficult to formulate using traditional approaches; for this reason, the micronized and ultramicronized forms have a reduced particle size compared to the native molecule, along with a greater bioavailability. Additionally, it seems that the association with flavones stabilizes the two molecules and enhances their pharmacological activities [256].

In particular, the association of these two molecules has been evaluated in a lot of different experimental models, such as in Alzheimer’s and Parkinson’s disease, vascular dementia, anxiety and depression, brain and spinal cord injury, and arthritis [254,255,256,257,258,259,260,261,262,263]. In these studies, it was proved that the association of PEA with luteolin was able to significantly decrease the neuroinflammatory and apoptotic pathways, modulating the cytokines release and the activation of astrocytes and microglia, decreasing the oxidative and nitrosative stress, and was able to enhance the expression of the neurotrophic factors promoting neuronal regeneration, as well as demonstrate that this association possesses the ability to modulate the autophagic process [254,255,256,257,258,259,260,261,262,263] (Table 4). 

The use of co-ultra PEALut was also evaluated in patients with autism disorders and in patients with stroke undergoing rehabilitation therapy [257,259]. Co-ultra PEALut treatment was tolerated well by the patients. Evaluation using the Autism Treatment Evaluation Checklist (ATEC) test revealed a decrease of scores, indicative of an increased behavioral outcome of about 23%. Co-ultra PEALut also reduced most indices of hyperactivity and improved the ability to understand simple commands and execute them. Unfortunately, no significant progress in speech profile has been observed yet [256]. 

In patients with stroke treated with co-ultra PEALut, the observations demonstrated a positive outcome in cognitive function, as well as in muscle spasticity and in mobility in daily living activities [258] (Table 5). 

## 13. Future Directions

Neuroinflammatory processes represent an important component in several diseases. Understanding the mechanisms that relate to systemic inflammation, even at a low-grade level, regarding neuroinflammation is an interesting area for research. The very early, possibly preclinical, stages of many pathologies could be the best window for therapeutic approaches or preventive interventions, which act on modifiable factors, such as systemic inflammation. Several studies have classified neuroinflammation as one of the driving forces for neurodegeneration. For example, glia activation, with the consequent release of pro-inflammatory mediators, is an important goal for diagnostic and therapeutic approaches in research. It would be fascinating to know whether activation of microglia revealed in the earliest phases of the disease is due to the anti-inflammatory M2 phenotype, later switching to the M1 phenotype, leading to further neuron damage. Once recognized, a clear panel of pro-inflammatory mediators could be added to the existing biomarker pool and used in the diagnostic process.

The influence of systemic inflammation on the CNS has been evident in acute episodes when the inflammatory status determines sickness behavior. A chronic inflammatory status appears to be responsible for the activation of glial cells, triggering a more persistent inflammatory status, leading to neuronal damage and neurodegeneration. Further studies are needed to understand the communication between the periphery and the CNS better, and to direct diagnostic evaluations, follow-up programs, and therapeutic and preventative approaches. 

The growing understanding that central immune mediators are important in controlling physiological activities of the CNS has updated the field of neuroimmunology. Both microglia and cytokines have been connected to the regulation of neurodevelopment, neuronal wiring, and synaptic plasticity. The functional significance and underlying mechanisms of these non-immunological functions persist unknown and await additional study. It is well-defined that the reductive conception of microglia as basically central immune cells is too simplistic. Reasonably, they appear as a different but heterogeneous cell population of the CNS with a high degree of functional diversity and complexity. Implying that modifications in microglia activity profiles and/or inflammatory factors with ongoing neuroinflammation may therefore be too simplistic and could result in misconceptions. In contrast, changes in neuroimmune systems should be deduced in relation to the functional complexity of immune cells and molecules in physiological brain processes.

## 14. Conclusions

As summarized in this review, fitting the endogenous neuroinflammatory regulators may represent a valid therapeutic strategy against the disorders of the central nervous system by targeting non-neuronal cells. In this context, the capacity of PEA to modulate several protective responses in inflammatory and pain conditions show that endogenously produced PEA may be an element of the homeostatic system that is able to control the inflammatory process. The findings obtained show that the treatment of disorders of the nervous system must be based on endogenous control of inflammatory processes. In this context, the role of the PEA that modulates the activity of both mast cells and glial cells contributes to the resolution of neuroinflammatory processes.

Clinical studies demonstrate the ability of PEA in micronized and ultramicronized forms to counter the painful symptoms of the electrophysiological alterations present in different pathologies that involve the peripheral and/or central nervous system, which are sustained due to excessive mast cell activation and almost always associated with microglia. Moreover, PEA can improve the alterations that characterize many neurological diseases. Several studies also confirmed the excellent tolerability of products containing co-ultra PEALut, PEA-m, and PEA-um, even when administered in combination with anti-inflammatory and analgesic/anticonvulsant drugs. 

These results suggest the use of PEA in micronized and ultramicronized forms as an innovative therapeutic tool for the treatment of all conditions characterized by the presence of neuroinflammatory processes and chronic painful states.

## Figures and Tables

**Table 1:** Publications in 2019 about the relationship between neuroinflammation and neurodegenerative disorders.

Pathology	References
Vascular Dementia	[12,13,14,15,16,17,18]
Depression	[19,20,21,22,23,24,25,26,27,28,29,30,31,32,33,34,35,36,37,38,39,40,41,42,43,44,45,46,47,48]
Alzheimer’s Disease	[49,50,51,52,53,54,55,56,57,58,59,60,61,62,63,64,65]
Parkinson’s Disease	[53,63,65,66,67,68,69,70,71,72,73,74,75,76,77,78,79,80,81,82]
Schizophrenia	[21,37,72,83,84,85,86,87,88,89,90]
Epilepsy	[21,37,72,83,84,85,86,87,88,89,90]

**Table 2:** Preclinical studies reporting efficacy of PEA.

Model	Animals	Effects	References
Visceral inflammatory pain	Rats	Decrease in NO production and neutrophil influx	[187]
Traumatic brain injury	Mice	Improves neurological, emotional, and biochemical outcomes	[188]
Spinal cord injury	Mice	Reduce mast cell activation and infiltration	[189]
Spinal cord injury	Mice	Reduce inflammatory markers	[190]
Parkinson’s disease	Mice	Reduce neuroinflammation	[193]
Colitis	Mice	Normalize the functional post-inflammatory accelerated intestinal transit	[192]
Colitis	Mice	Improve symptoms of colitis	[191]
Dermatitis	Dogs	Reduce pruritus and skin lesions	[194]
Contrast-agent-induced nephropathy	Rats	Prevent nephropathy in and alteration of biochemical parameters	[195]
Idiopathic pulmonary fibrosis	Mice	Reduces lung inflammation	[196]
Tibia fracture model	Mice	Decrease mast cell density, nerve growth factor, matrix metalloproteinase 9, and cytokines expression	[197]
Alzheimer’s disease	Mice	Normalize astrocytic function, rebalance glutamatergic transmission, and restrain neuroinflammation	[198]
Joint pain	Rats	Reduce igeminal nerve sensitization	[199]

**Table 3:** Clinical studies reporting efficacy of PEA.

Pathology	Effects	References
Lombosciatalgia	Analgesic effect	[210]
Lombosciatalgia	Analgesic effect	[211]
Chronic pain	Analgesic effect	[209]
Temporomandibular joint pain	Analgesic effect	[212]
Peripheral diabetic neuropathy	Reduce pain symptoms characteristic of diabetic neuropathy	[213]
Carpal tunnel syndrome	Improve sleep quality, confirming a correlation between sleep disorders and pain intensity	[208]

**Table 4:** Preclinical studies reporting the efficacy of PEA + luteolin (PEALut).

Model	Animals	Effects	References
Sci	Mice	Stimulate hippocampal neurogenesis and dendritic spine maturation	[257]
Paw edema	Rats	Reduce inflammation and pain	[256]
Alzheimer’s disease	Cell culture	Reduce inducible nitric oxide synthase and GFAP expression, restore neuronal NO synthase and BDNF, and reduce the apoptosis	[261]
Parkinson’s disease	Mice	Modulate the neuroinflammatory process and the autophagic pathway	[259]
Anxiety and depression	Mice	Antidepressant effect	[254]
Spinal Cord Injury	Mice and cell culture	Improve motor activity, reduced cyclooxygenase-2 (cox-2), and inducible nitric oxide synthase (inos)	[262]
Autism	Mice	Ameliorate social and nonsocial behaviors	[256]
Stroke	Rats	Reduce neuroinflammation	[259]
Spinal Cord Injury	Mice	Promote neuronal regeneration	[257]

Sci: Spinal cord injury.

**Table 5:** Clinical studies reporting the efficacy of PEALut.

Pathology	Effects	References
Autism	Reduction in stereotypes	[256]
Stroke	Improve clinical outcome	[258]
